# Reciprocal Interplay Between Astrocytes and CD4+ Cells Affects Blood-Brain Barrier and Neuronal Function in Response to β Amyloid

**DOI:** 10.3389/fnmol.2020.00120

**Published:** 2020-07-03

**Authors:** Simona Federica Spampinato, Sara Merlo, Evelina Fagone, Mary Fruciano, Yasuteru Sano, Takashi Kanda, Maria Angela Sortino

**Affiliations:** ^1^Section of Pharmacology, Department of Biomedical and Biotechnological Sciences, University of Catania, Catania, Italy; ^2^Department of Clinical and Experimental Medicine, University of Catania, Catania, Italy; ^3^Department of Neurology and Clinical Neuroscience, Yamaguchi University Graduate School of Medicine, Ube, Japan

**Keywords:** BBB, Th2, IL-4, BDNF, synaptophysin, neurodegeneration, neuroinflammation, Alzheimer’s disease

## Abstract

**Background**: In Alzheimer’s disease (AD) neuronal degeneration is associated with gliosis and infiltration of peripheral blood mononuclear cells (PBMCs), which participate in neuroinflammation. Defects at the blood-brain barrier (BBB) facilitate PBMCs migration towards the central nervous system (CNS) and in particular CD4+ T cells have been found in areas severely affected in AD. However, the role of T cells, once they migrate into the CNS, is not well defined. CD4+ cells interact with astrocytes able to release several factors and cytokines that can modulate T cell polarization; similarly, astrocytic properties are modulated after interaction with T cells.

**Methods**: In *in vitro* models, astrocytes were primed with β-amyloid (Aβ; 2.5 μM, 5 h) and then co-cultured with magnetically isolated CD4+ cells. Cytokines expression was evaluated both in co-cultured CD4+ cells and astrocytes. The effects of this crosstalk were further evaluated by co-culturing CD4+ cells with the neuronal-like SH-SY5Y cell line and astrocytes with endothelial cells.

**Results**: The pattern of cytokines and trophic factors expressed by CD4+ cells were strongly modulated in the presence of Aβ-primed astrocytes. Specifically, the percentage of IL-4+ and IFNγ+ CD4+ cells was significantly increased and reduced, respectively. Further, increased BDNF mRNA levels were observed in CD4+ cells. When SH-SY5Y cells were co-cultured with astrocyte-conditioned CD4+ cells and exposed to Aβ, the reduction of the presynaptic protein synaptophysin was prevented with a BDNF-dependent mechanism. In astrocytes co-cultured with CD4+ cells, reduced mRNA levels of inflammatory cytokines and VEGF were observed. This was paralleled by the prevention of the reduction of claudin-5 when astrocytes were co-cultured with endothelial cells.

**Conclusion**: Following Aβ exposure, there exists reciprocal crosstalk between infiltrating peripheral cells and astrocytes that in turn affects not only endothelial function and thus BBB properties, but also neuronal behavior. Since astrocytes are the first cells that lymphocytes interact with and are among the principal players in neuroinflammation occurring in AD, understanding this crosstalk may disclose new potential targets of intervention in the treatment of neurodegeneration.

## Introduction

Alzheimer’s disease (AD) is a neurodegenerative disorder whose typical pathological hallmarks are β-amyloid (Aβ) plaques and neurofibrillary tangles. The neuronal degeneration that characterizes the disease is associated with microgliosis and astrocytosis, causing an inflammatory state that has been described in the brain of AD patients. Peripheral inflammation can affect the central nervous system (CNS) and increased infiltration of immune cells in the brain of AD patients and corresponding animal models has been reported (Togo et al., 2002; Michaud et al., 2013; Zenaro et al., 2015). Also, in particular in AD patients, changes in the distribution of monocytes and lymphocytes as well as cytokines production have been described (Pellicano et al., 2012; Zhang et al., 2013). Enhanced blood-brain barrier (BBB) leakage and endothelial overexpression of adhesion molecules (such as vascular cell adhesion molecule 1, VCAM-1, and intercellular adhesion molecule-1, ICAM-1) induce leukocytes migration into the CNS close to Aβ plaques (Zenaro et al., 2015). Above the others, migration of both CD4+ and CD8+ T cells into the CNS of AD patients is significantly increased and observed mainly in areas that are typically affected in AD (Togo et al., 2002). However, the role of T cells, once they migrate through the BBB, is still not completely clarified. CD4+ subsets may differently influence CNS responses. T-helper (Th)1 and Th17 infiltration causes increased microglial activation and Aβ load (Browne et al., 2013) and enhanced neuronal degeneration through the release of specific interleukins (IL-1β, -6, -17, -21 and -22; Zhang et al., 2013). In contrast, Th2, that above the others, release factors like IL-4, IL-10 and brain-derived neurotrophic factor (BDNF; Eremenko et al., 2019), associate with decreased pathological exacerbation (Cao et al., 2009) and reduce glial response to inflammatory cytokines (McQuillan et al., 2010).

Astrocytes support neurons, but their involvement in CNS immunity has been underestimated. For their physical association with endothelial cells at the BBB, they are indeed the first cell type facing infiltrating cells (Iadecola and Nedergaard, 2007). They are directly involved in innate CNS immunity (Farina et al., 2007), but their ability to prime T cells seems relatively weak if compared to dendritic cells or microglia (McQuillan et al., 2010). Astrocytes further support endothelial cells function at the BBB, physically and through the release of several factors, including growth factors, such as vascular endothelial growth factor (VEGF), and inflammatory interleukins (IL-1β and IL-6) that may modulate the response of the barrier both in physiological and pathological conditions (Spampinato et al., 2019a).

Starting from the interaction between T cells and astrocytes, the aim of our study was then to analyze, in an *in vitro* system based on independent cellular cultures, the reciprocal interplay among infiltrating peripheral T cells, CNS resident cells, including astrocytes and neurons, and endothelial cells and to establish whether this crosstalk can be modified when the different cell types are exposed to Aβ.

## Materials and Methods

### Reagent

All cell culture plastics were from BD Falcon. Polycarbonate membrane transwell inserts (0.4, μm pores, no. 353090 and 8 μm pores no. 3422), collagen I rat tail (no. 354236) and lymphocyte separation medium (no. 25-072-cv) were provided by Corning. β-amyloid 1–42 peptide (Aβ; Innovagen, no. SP-BA42-1) was solubilized in dimethylsulfoxide as a 5 mM stock solution. Subsequent dilutions were made in the medium. A concentrated solution of Aβ 100 μM was aggregated by overnight incubation at room temperature, followed by freeze-thaw cycles for enrichment in oligomers, as previously described (Merlo and Sortino, 2012). For experiments, Aβ (1–42) was diluted in culture medium to a final concentration of 2.5 μM. The state of oligomerization of the peptide was evaluated by western blot analysis showing a mixture of monomers, dimers, tetramers, and different size oligomers, as previously shown (Merlo and Sortino, 2012). Human recombinant brain-derived neurotrophic factor (BDNF, no. 450-02) and human recombinant interleukin 4 (IL-4, no. 200-04) were from Peprotech Inc. The selective TrkB antagonist ANA-12 was provided by Sigma-Aldrich (no. 5063040001).

### Cell Cultures

TY-10 cells, brain microvascular endothelial cells, and hAST, astrocytic cells, are adult human immortalized cell lines, transfected with a plasmid expressing temperature-sensitive Simian virus-40 large T-antigen (ts-SV40-LT) and the catalytic subunit of human telomerase, as previously described (Haruki et al., 2013). Both cell lines were developed at Yamaguchi University (Japan), in the labs of Dr. Sano and Kanda. TY-10 cells were grown in MCDB-131 media (Sigma–Aldrich, no. 10372019) supplemented with EGM-2 SingleQuots (Lonza, no. LOCC4176) and 20% heat-inactivated fetal bovine serum (FBS, Thermo Fisher Scientific). hAST were grown in astrocyte medium containing 2% heat-inactivated FBS, astrocyte growth supplement, and penicillin/streptomycin (P/S) solution, as provided with the Astrocyte media kit (ScienCell Research Laboratories, no. 1801-SC). For experiments, both TY-10 and hAST cells were grown at 33°C for 2 days and then transferred to 37°C, where they exhibited growth arrest and differentiation. After differentiation for 2 days at 37°C, cells were exposed to Aβ. The continuous human neuroblastoma cell line, SH-SY5Y cells, were grown in DMEM/F12 medium (ThermoFisher Scientific, no. 21331-020) supplemented with 10% FBS and P/S. The amount of serum in the medium was progressively reduced to 1% to allow differentiation. The protocol here described was set in our lab and lasted 5 DIV. Gradual serum reduction induced cell cycle arrest and neuronal differentiation. The reduction of neuronal-like cell proliferation in these conditions was confirmed by cytofluorometric analysis of cell cycle distribution following propidium iodide incorporation, as previously demonstrated (Merlo et al., 2018). Experiments were performed in DMEM/F12 supplemented with 1% FBS. Peripheral blood mononuclear cells (PBMCs) were isolated from fresh heparinized blood of healthy subjects by density centrifugation with Lymphocyte Separation Medium (Corning, Thermo Fisher Scientific), as previously described (Man et al., 2008). Blood was from de-identified subjects donating to the Hospital blood bank for transfusion purposes. This exempted the study from Ethics Committee authorization. We used buffy coats derived from six healthy donors for the transmigration assay, from eight healthy donors to evaluate CD4 polarization and from seven healthy donors for CD4/SH-SY5Y co-cultures. For the transmigration assay, PBMCs were resuspended in transendothelial migration (TEM) buffer (RPMI 1640 without phenol red, 1% bovine serum albumin, Hepes, L-glutamine, Na-pyruvate, MEM non-essential amino acids, all from Thermo Fisher Scientific). CD4+ cells were negatively selected using the CD4^+^ T Cell Isolation Kit (MiltenyiBiotec, no. 130-096-533) and grown in phenol red-free RPMI medium supplemented with FBS 10%, glutamine and non-essential amino acids, either alone or in co-culture with astrocytes.

### Co-cultures of Astrocytes and CD4+ Cells and SH-SY5Y Cells and CD4+ Cells

Co-cultures were set on culture transwell inserts, allowing the passage of cytokines and growth factors, but no direct physical contact between different cell lines. Astrocytes (3 × 10^5^ cells per wells) were plated on six multiwell dish plates, grown in astrocyte medium for 2 days at 33°C, and then kept for 2 days at 37°C. Aβ (2.5 μM) treatment was performed in astrocyte medium for 5 h at 37°C, the time point we chose to induce an early astrocytic response to Aβ, as already observed (Spampinato et al., 2019b). CD4+ cells (1 × 10^6^ cells per wells) were transferred on culture transwell inserts with 0.4 μm pore (Falcon). At the time of co-culture, the ratio astrocytes/CD4 was 1:2. Co-cultures were maintained for 18/48 h, according to different experimental settings, grown in phenol red-free RPMI medium supplemented with 10% FBS, glutamine and non-essential amino acids (all from Thermo Fisher Scientific). To evaluate variations in mRNA levels, CD4+ cells and astrocytes co-cultured for 18 h were collected and pellets were processed for RT-PCR analysis. After 48 h in co-culture, a longer time point chosen to evaluate cytokine expression, CD4+ cells were collected and counted and an aliquot was fixed and processed for flow cytometry. Remaining cells were co-cultured with SH-SY5Y neuronal-like cells as follows. SH-SY5Y (4.5 × 10^5^ cells per wells) were plated on 12 multiwell dish plates and progressively deprived of serum to allow differentiation. After 5 DIV, CD4+ cells (1.5 × 10^6^ cells per wells) were transferred on culture transwell inserts with 0.4 μm pore, and the ratio between SH-SY5Y/CD4 at the time of co-culture was 1:2,5. Co-cultures were exposed to Aβ (2.5 μM) for 24 h, and then SH-SY5Y cells were processed for western blot analysis.

### Migration Assay

The protocol for static transmigration assay has been previously described (Spampinato et al., 2019b). For the static transmigration assay, 6.5 mm polycarbonate membrane cell culture inserts with 8.0 μm *pore* (Corning^®^ Transwell^®^) were used. hAST (3 × 10^5^ cells per wells) were seeded on the abluminal side of the membrane, and after attachment, inserts were flipped and TY-10 (5 × 10^5^ per membrane, with a ratio astrocytes/endothelial cells of 1:1,6), seeded on the luminal side. This setting allowed the passage of soluble factors between endothelial cells and astrocytes layer, but not their direct physical contact. Co-cultures were grown in astrocyte medium for 2 days at 33°C and then kept for 2 days at 37°C. Aβ (2.5 μM) treatment was performed in astrocyte medium for 18 h at 37°C, a time point at which Aβ exposure induces PBMCs migration through the endothelial barrier (Spampinato et al., 2019b). At this time point, the ratio astrocytes/endothelial cells was 1:1,7. Before the assay, the apical endothelial layer was exposed to CXCL12 (50 ng/ml in TEM buffer, Peprotech, no. 300-28A) and incubated for 15 min at 37°C. FBS 1% was used as chemoattractant in the abluminal side. PBMCs (2.8 × 10^6^ cells per assay, with a ratio of endothelial cells/PBMCs of 1:3, six at the time of co-culture) were added on the top of the endothelial layer. The assay was ended after a total of 18 h. Migrated PBMCs were recovered from the bottom chamber and counted.

### Western Blot

After treatments, pellets were collected and lysed in RIPA lysis buffer (Sigma-Aldrich, no. R0278) supplemented with protease and phosphatase inhibitors. Thirty micrograms of each sample were separated by sodium dodecyl sulfate page and transferred to nitrocellulose membranes. Membranes were blocked with Blocker^TM^ FL Fluorescent Blocking Buffer (Thermo Fisher Scientific, no. 37565) and probed with the following primary antibodies overnight: anti-rabbit Claudin-5 (1:300; Thermo Fisher Scientific, no. 34-1600), anti-mouse ICAM-1 (1:800; Santa Cruz Biotechnology, no. SC-8439), anti-mouse GAPDH (1:800; Millipore, no. MAB 374); anti-mouse synaptophysin (1:5,000, Santa Cruz Biotechnology no. SC-17750). Membranes were then processed for immunodetection using specific fluorescent AlexaFluor^®^ 647 and AlexaFluor^®^ 488 Plus-conjugated secondary antibodies. Fluorescent signals were detected using IBright 1500 (Thermo Fisher Scientific). Band intensity was analyzed using the image processing software “ImageJ” developed by NIH and in the public domain.

### Flow Cytometry

CD4+ cells were either cultured alone and exposed to Aβ 2.5 μm for 48 h or co-cultured for 48 h with astrocytes previously exposed to Aβ for 5 h. Both CD4+ cells and astrocytes were then collected and fixed using the Inside stain kit (Miltenyi Biotec, no. 130-090-477), following the manufacturer protocols and stained using either anti-human IL4-phycoerythrin (PE) antibody (1 h at RT, 1:50 Miltenyi Biotec, no. 130-091-647) or mouse anti-human interferon (IFN)-γ (overnight at 4°C, 1:120, Thermo Fisher Scientific no. 710287), followed by staining with the secondary anti-mouse-PE antibody (1:500, 1 h at RT). The samples were examined using Amnis^®^ ImageStream^®^ (Millipore), and data were analyzed using the Amnis Ideas^®^ software.

### Quantitative Real-Time Polymerase Chain Reaction (PCR)

CD4+ cells were either cultured alone and exposed to Aβ 2.5 μm or co-cultured for 18 h with astrocytes previously exposed to Aβ for 5 h. Both CD4+ cells and astrocytes were then collected and total RNA was extracted from cell cultures using the RNeasy Plus Mini Kit or Micro Kit (Qiagen, no. 74134). One microgram of RNA was used for cDNA synthesis, using the Superscript-VILO kit (Thermo Fisher Scientific). Quantitative RT-PCR was performed with Rotor-Gene Q using QuantiNova SYBR Green PCR Kit (Qiagen, no. 208054). The melting curves obtained after each PCR amplification reaction confirmed the specificity of the 2-[N-(3-dimethylaminopropyl)-N-propylamino]-4-[2,3-dihydro-3-methyl-(benzo-1,3-thiazol-2-yl)-methyli-dene]-1-phenyl-quinolinium (SYBR Green assays). The following Quantitec primers (Qiagen) were used: IL-6 (QT00083720), IL-1β (QT00021385), IL-4 (QT00012565), vascular endothelial growth factor (VEGF)-A (QT01010184) and human RPLP0 (QT00075012) as an endogenous control. Expression fold changes were calculated by applying the 2^−ΔCt^ method.

### Statistical Analysis

All data are expressed as means ± SEM of 3–10 different experiments each run in duplicates or triplicates as specified in the figure legends. Intra assay variability was always less than 5%. Data were analyzed by one-way ANOVA, followed by Newman–Keuls test for significance. *p* < 0.05 was taken as the criterion for statistical significance when three or more conditions were compared. Student’s *t*-test was applied between two groups. *p* < 0.05 was taken as the criterion for statistical significance.

## Results

### CD4+ Cells Cross the BBB in Response to Aβ

**Figure 1:**
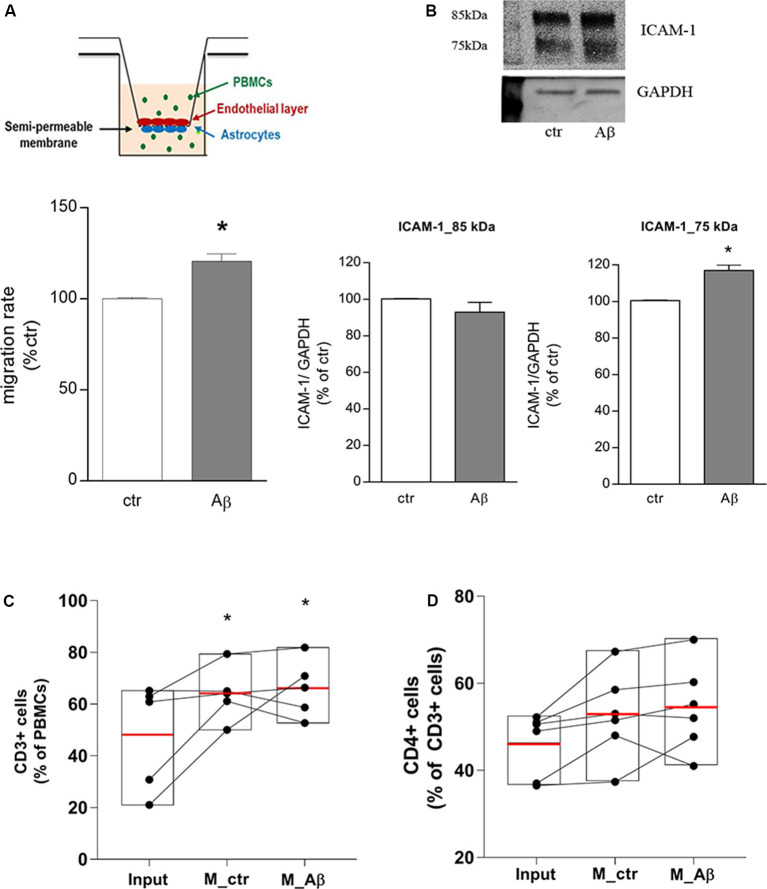
Aβ induces CD3+/CD4+ cells transmigration through an *in vitro* blood-brain barrier (BBB) model. Endothelial/astrocytes co-cultures were exposed to Aβ (2.5 μM) for 18 h and the number of PBMCs migrating through the barrier was evaluated after an additional 18 h. PBMCs migration represents the ratio of migrated cells through the barrier, given a constant input, expressed as a percentage of migration in control conditions **(A)**. Protein expression of ICAM-1 in endothelial cells co-cultured with astrocytes and exposed to Aβ (2.5 μM) for 18 h was evaluated by western blot analysis. Densitometric analyses of the two isoform bands (85 and 75 kDa) and a representative blot are reported **(B)**. PBMCs migrated through the *in vitro* BBB were immunostained for the T cell co-receptor CD3 **(C)** and CD4 **(D)** and processed through flow cytometry analysis. Boxplots for total cells (input), cells migrated under control conditions (M_ctr), and cells migrated after treatment of astrocytes/endothelial cells with Aβ (2.5 μM) for 18 h (M_Aβ) are shown. Data are mean ± SEM of four **(A)** to six **(B–D)** independent experiments. *p < 0.05 vs. control, ctr **(A)** and input **(C)**. Significance was assessed by Student’s *t*-test **(A,B)** and by one-way ANOVA followed by Newman–Keuls test **(C,D)**.

Endothelial/astrocyte co-cultures were exposed to Aβ 2.5 μM for 18 h. Freshly isolated PBMCs from healthy donors were used to evaluate the capability of PBMCs to migrate through the *in vitro* BBB model. PBMCs migration rate through the barrier was significantly induced after 18 h of Aβ exposure (Figure 1A); this effect was accompanied by endothelial overexpression of the lower MW ICAM-1 glycoform (75 kDa), tightly involved in TEM (Spampinato et al., 2019b), as shown by western blot analysis on endothelial extracts (Figure 1B). At the end of the assay, PBMCs migrated through the *in vitro* barrier were recovered, enumerated, and analyzed by flow cytometry to evaluate their cellular subsets. Compared to PBMCs not subjected to the assay and maintained under the same conditions (input), the population of migrated cells was enriched in the percentage of CD3+ cells (Figure 1C). About 50% (53.5 ± 2.8%) of the migrated CD3+ population was represented by CD4+ cells, suggesting, as a consequence, a slight enrichment in the population of CD4+ cells crossing the *in vitro* barrier. However, direct analysis of the CD4+ cells within the CD3+ population showed only a trend toward an increase, without yielding statistical significance (Figure 1D).

### Aβ Through Astrocytes Modifies CD4+ Cell Polarization

**Figure 2:**
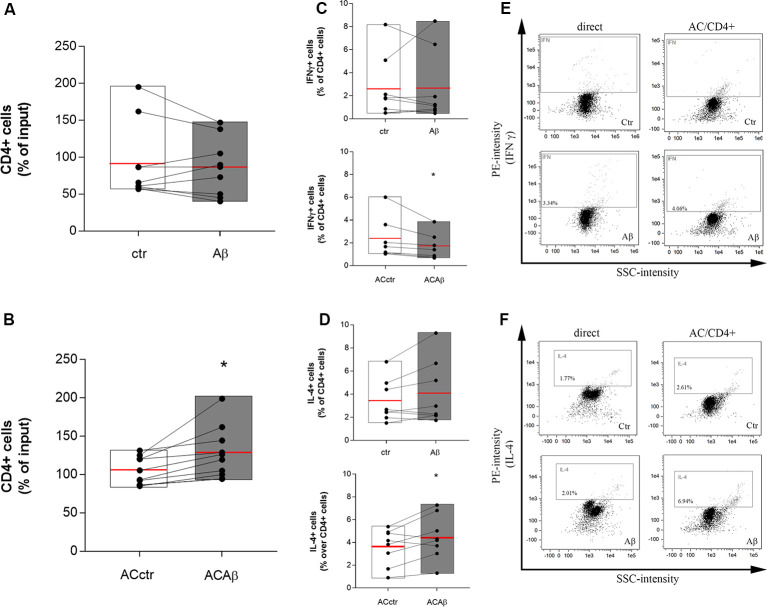
Aβ treated astrocytes support CD4+ cell survival and skew their phenotype. CD4+ cells were exposed either directly to Aβ (2.5 μM) **(A)** or co-cultured with control (ACctr) or Aβ pre-exposed astrocytes (ACAβ) **(B)** for 48 h. After treatments, CD4+ cells were collected and enumerated. Given a constant input, values express the percentage of viable cells counted vs. the input. Through cytofluorometric analysis the expression of IFNγ **(C,E)** and IL-4 **(D,F)** was evaluated in CD4+ cells exposed either directly to Aβ (2.5 μM, **C,D**, upper panels) or to control (ACctr) or Aβ-pretreated astrocytes (ACAβ; **C,D**, lower panels) for 48 h. Representative plots of flow cytometric analysis for IFNγ **(E)** and IL-4 **(F)** are reported. Data are mean ± SEM of six **(A,B)** or eight **(C,D)** independent experiments. *p < 0.05 vs control (ctr) Significance was assessed by Student’s *t*-test.

CD4+ cells were isolated from freshly prepared PBMCs and co-cultured with either control astrocytes (ACctr) or astrocytes primed with Aβ for 5 h (2.5 μM, ACAβ). The 5 h pretreatment time and the Aβ concentration were chosen since, in our experience, responses to Aβ are already measurable at these conditions, without affecting endothelial and astrocytic viability (Spampinato et al., 2019b). Astrocytes/CD4+ co-cultures were maintained for 48 h. Simultaneously, CD4+ cells were directly exposed to Aβ (2.5 μM). At the end of the incubation, CD4+ cells were collected and counted before being fixed and processed for flow cytometry. The 48 h time point was chosen because, according to preliminary data (not shown), a 24 h astrocytes/CD4 co-culture was not sufficient to induce any modifications in the expression and storage of cytokines in CD4+ cells. Aβ exposure did not modify *per se* the total number of CD4+ cells (Figure 2A). However, when co-cultured with ACAβ, CD4+ cell number increased (Figure 2B). Once collected, CD4+ cells, directly exposed to Aβ (2.5 μM) or co-cultured with ACctr or ACAβ for 48 h, were processed for flow cytometry. The expression of specific markers for both Th1 and Th2 cells (IFNγ and IL-4, respectively) was evaluated. The expression of Foxp3+ cells in our system was very limited, thus Treg polarization was not evaluated. When directly exposed to Aβ, the percentage of CD4+ cells expressing IFNγ or IL-4 was not affected (Figures 2C,D, upper panels). In contrast, when CD4+ cells were co-cultured with Aβ-pretreated astrocytes (ACAβ), the percentage of IFNγ+ cells was reduced (Figure 2C, lower panel) and accordingly, the percentage of IL-4+ cells significantly increased (Figure 2D lower panel). Representative plots of flow cytometric analysis are reported in Figures 2E,F.

### CD4+ Cells Cultured With Aβ-Pretreated Astrocytes Modify the Expression of Synaptic Proteins

**Figure 3:**
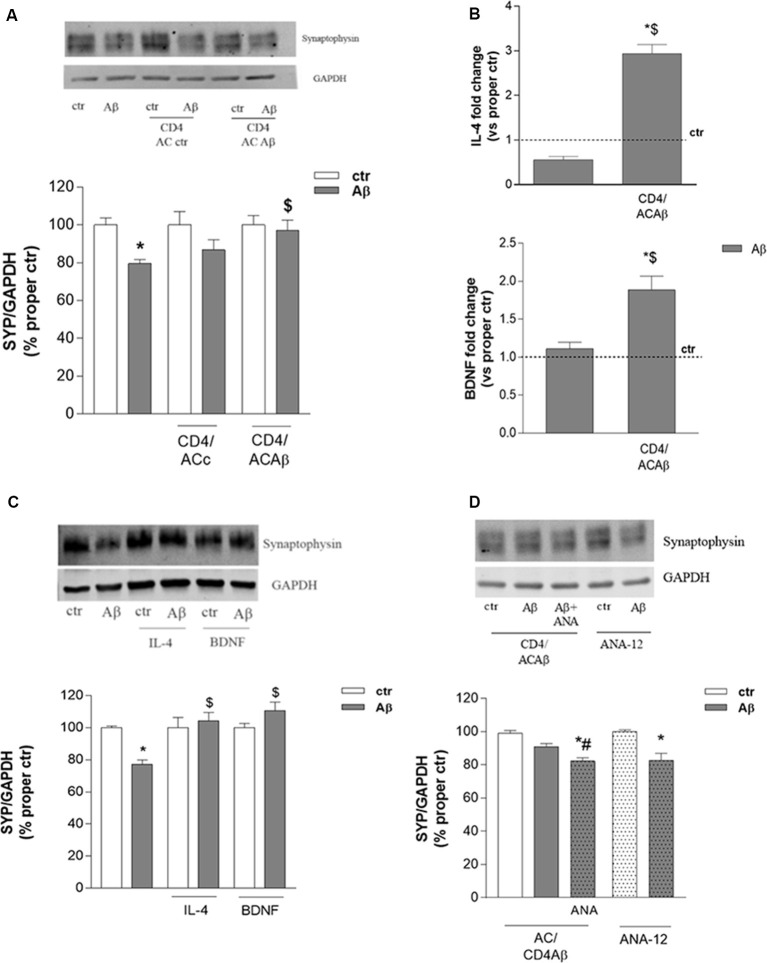
CD4+ cells co-cultured with Aβ-treated astrocytes express BDNF and prevent Aβ-induced changes in synaptic protein expression. The expression of the synaptic protein synaptophysin was evaluated by western blot in SH-SY5Y cells treated with Aβ (2.5 μM) for 24 h either alone or in the presence of CD4+ cells previously co-cultured with astrocytes under control (ACctr) or Aβ-treated conditions **(A)**. IL-4 and BDNF mRNA levels were evaluated in CD4+ cells exposed to Aβ (2.5 μM) either directly or in co-culture with astrocytes (CD4/ACAβ) for 18 h **(B)**. Data are reported as fold change vs. proper control. Expression of synaptophysin in SH-SY5Y cells treated with Aβ (2.5 μM) alone or in co-treatment with IL-4 (10 ng/ml) and BDNF (10 ng/ml) are reported in **(C)**. Expression of synaptophysin under conditions described in A in the presence of the TrkB antagonist ANA-12 (20 μM) added 30 min before Aβ (2.5 μM, 24 h; CD4/ACAβ) **(D)**. Representative blots are reported in **(A,C,D)**. Data are mean ± SEM of six **(A)**, four **(C)**, and three **(B,D)** independent experiments. *p < 0.05 vs. control, ctr. ^$^*p* < 0.05 vs. direct Aβ. ^#^*p* < 0.05 vs. Aβ alone. Significance was assessed by one-way ANOVA followed by Newman–Keuls test.

CD4+ cells, cultured for 48 h with either control astrocytes (CD4/ACctr) or Aβ-treated astrocytes (CD4/ACAβ), were then transferred on top of an insert and co-cultured with differentiated human neuronal-like SH-SY5Y cells. Co-cultures were exposed to Aβ (2.5 μM) for 24 h and neuronal damage was assessed by evaluating the expression of the presynaptic protein synaptophysin. As expected, the expression of synaptophysin was significantly reduced by 24 h treatment with Aβ (2.5 μM). This effect was not modified in the presence of CD4/ACctr (Figure 3A). In contrast, Aβ exposure affected only slightly the expression of synaptophysin when SH-SY5Y cells were co-cultured with CD4+ cells polarized in the presence of Aβ-pretreated astrocytes (CD4/ACAβ, Figure 3A). To establish whether the observed effects on expression of the synaptic protein were due to changes of CD4+ cells, we investigated their expression of IL-4 and BDNF. When co-cultured for 18 h with Aβ-pretreated astrocytes (ACAβ), IL-4 and BDNF mRNA levels were significantly increased in CD4+ cells compared to those cultured in the presence of control astrocytes (ACctr). No changes of IL-4 and BDNF mRNA levels were observed when CD4+ cells were directly exposed to Aβ, whereas they were significantly increased in the presence of Aβ-pretreated astrocytes (ACAβ, Figure 3B). To ascertain whether BDNF and IL-4 could indeed affect neuronal response to Aβ, we treated SH-SY5Y cells in the presence of exogenously added IL-4 (10 ng/ml) and BDNF (10 ng/ml) for 24 h and analyzed the expression of synaptophysin. Both treatments prevented the reduced expression of synaptophysin induced by Aβ (Figure 3C).

Interestingly, prevention of Aβ-induced synaptophysin reduction in the presence of CD4/ACAβ was not observed any more on SH-SY5Y cells treated with the BDNF receptor TrkB selective antagonist ANA-12, known to counteract, *in vitro*, BDNF functions at the concentration of 20 μM (Merlo et al., 2018, Figure 3D). In contrast, when added directly to SH-SY5Y cells, ANA-12 did not modify Aβ effects on synaptophysin expression (Figure 3D).

### CD4+ Cells Interfere With Cytokine Expression in Astrocytes and Modify BBB Function

**Figure 4:**
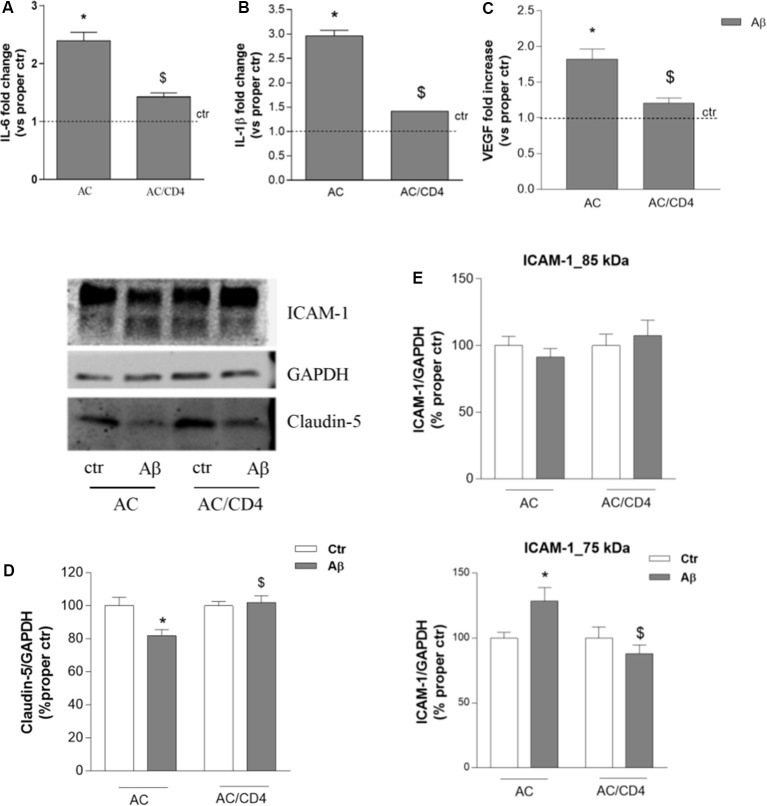
CD4+ cells modulate astrocyte ability to affect endothelial properties. Astrocytes (AC) were pre-exposed to Aβ (2.5 μM) for 5 h and then co-cultured with CD4+ cells (AC/CD4). Astrocytic mRNA levels of IL-6 **(A)**, IL-1β **(B)**, and VEGF **(C)** were evaluated by RT-PCR. Endothelial cells were co-cultured with naïve astrocytes (AC) or astrocytes co-cultured with CD4+ cells (AC/CD4) and treated with Aβ (2.5 μM) for 18 h. Protein expression of claudin-5 and ICAM-1 was evaluated by western blot analysis. A representative blot and densitometric analysis of claudin-5 **(D)** and the two ICAM-1 isoforms (85 and 75 kDa) are reported **(E)**. Data are mean ± SEM of three **(A–C)**, four **(D)**, and six **(E)** independent experiments. *p < 0.05 vs. control, ctr. ^$^*p* < 0.05 vs. the condition without CD4 (AC). Significance was assessed by one-way ANOVA followed by Newman–Keuls test.

We then evaluated whether and how CD4+ cells affected astrocytic response to Aβ. Gene expression of inflammatory cytokines (IL-1β and IL-6) was investigated in astrocytes pre-exposed to Aβ for 5 h and then cultured either alone (AC) or in the presence of CD4+ cells (AC/CD4) for further 18 h. Aβ induced enhanced expression of both IL-6 and IL-1β, as measured by RT-PCR, an effect prevented by pre-exposure to Aβ followed by co-culture with CD4+ cells (AC/CD4, Figures 4A,B). Also, under the same conditions, Aβ failed to induce enhanced VEGF expression in astrocytes co-cultured with CD4+ (Figure 4C). Astrocytes previously exposed to Aβ for 5 h and then cultured either alone or in the presence of CD4+ (AC/CD4) for 48 h, were co-cultured with endothelial cells and exposed to 2.5 μM Aβ for 24 h. Endothelial expression of claudin-5 and ICAM-1 was evaluated by western blot analysis. Aβ reduced claudin-5 expression, but this effect was prevented in endothelial cells co-cultured with AC/CD4 (Figure 4D). Modifications in ICAM-1 expression were observed only for the lower MW glycoform of the protein. In particular, ICAM-1 (75 kDa) was induced in endothelial/astrocytes co-cultures exposed to Aβ for 24 h, but not when astrocytes were previously exposed to CD4+ cells (Figure 4E).

## Discussion

During AD, in particular in the late stages of the disease, the BBB can be severely damaged, and thus the access to the CNS of solutes and immune cells is altered. T cells in particular may easily cross the damaged BBB in these conditions. In the cerebrospinal fluid and the brain of AD patients, the proportion of CD4+ and CD8+ T cells is elevated (Laurent et al., 2017). Accordingly, increased levels of peripheral T cells have been described in postmortem brains from AD patients, as a consequence of damaged BBB and increased release of chemokines and cytokines by degenerating and activated cells in the CNS (Mietelska-Porowska and Wojda, 2017). Once they cross the BBB, T cells immediately reach the glia *limitans*, and herein they interact with astrocytes. It has been widely reported that microglia are the main resident cells in the CNS and they can thus act as potential antigen-presenting cells. Astrocytes also express major histocompatibility complex (MHCI and II) and under inflammatory challenge, they overexpress the co-stimulatory factors CD80 and CD86 (reviewed in Xie and Yang, 2015), although their ability to prime T cells seems relatively weak if compared to dendritic cells or microglia (McQuillan et al., 2010). However, the interaction between astrocytes and CD4+ cells is not exclusively dependent on the contact between astrocytic MHC/T cell receptors. Astrocytes release several cytokines and chemokines that can potentially modify T cell functions. We first noticed that when co-cultured with astrocytes and in particular with Aβ-pretreated astrocytes, CD4+ cell number increased. We have not investigated what is at the basis of this effect, but it appears in accordance with what was previously observed in astrocytes/CD4 co-cultures, where glutamate released by astrocytes promoted CD4 cell division (Beurel et al., 2014).

CD4+ cells changed their response to Aβ when co-cultured with Aβ-pretreated astrocytes, as shown by reduced and increased levels of IFNγ and IL-4, respectively. Of note, only these CD4+ cells prevented the loss of neuronal synaptic function. When co-cultured with unstimulated astrocytes (ACctr), this effect was not observed, clearly indicating that Aβ modifies astrocytes which in turn affect CD4+ cells polarization toward a less inflammatory phenotype. Accordingly, it has been described that, when damage occurs in the CNS, astrocytes may stimulate the innate immune response skewing it towards the production of Th2 cytokines (Schmitz et al., 2005; Neill et al., 2010). In these conditions, the production of IL-4 is increased (Walsh et al., 2015), and infiltrating T cells appear to be the main source of this cytokine in the CNS, as both microglia and astrocytes release very low levels of IL-4 (Walsh et al., 2015). A protective role for IL-4 has been described in AD transgenic mice, where administration of this cytokine, in association with IL-13, improved cognitive function (Kiyota et al., 2010; Kawahara et al., 2012). Administration of amyloid-specific Th2 cells improved spatial memory, decreased microglial reactivity and reduced Aβ pathology in AD animal models (Cao et al., 2009), pointing out the role played by IL4-producing cells in reducing AD damage. Higher peripheral IL-4 concentration was found in MCI patients whereas increased disease severity seemed to be associated with reduced IL-4 levels (King et al., 2018). Finally, interacting with the neuronal IL-4 receptor, IL-4 could mediate a protective function (Steinman, 2015; Walsh et al., 2015), inducing actin modifications and axonal sprouting, as observed in experimental autoimmune encephalomyelitis (EAE) models (Vogelaar et al., 2018).

Together with the increased production of IL-4, CD4+ cells co-cultured with Aβ-pretreated astrocytes showed also an enhanced expression of BDNF. We did not measure IL-4 and BDNF released by astrocytes, but the increase of BDNF mRNA expression was remarkable and suggestive of an enhanced release, as previously described (Kerschensteiner et al., 1999). As for IL-4, the increase of its mRNA expression was paralleled by enhanced intracellular content as by flow cytometric analysis. We focused our attention on BDNF, a known regulator of the expression, function and localization of presynaptic protein synaptophysin, as our marker of choice to evaluate neuronal damage (Tartaglia et al., 2001; Bamji et al., 2006; Zhang et al., 2017). Of note, partial preservation of pre-synaptic function observed with CD4+ cells pre-exposed to Aβ-treated astrocytes was blunted, at least in part, under conditions of a blockade of the BDNF receptor TrkB, confirming the main role for the growth factor in the observed effect. This is not surprising since the role of BDNF in preserving neuronal function is largely proved. Increased BDNF serum levels are associated with reduced cognitive decline (Laske et al., 2011), while BDNF therapy results in increased synaptic efficiency and plasticity (Murer et al., 2001; Nagahara et al., 2009; Budni et al., 2015) and increased neuronal survival (Arancibia et al., 2008). What probably appears unexpected is that CD4+ cells are the main source of BDNF. However, supportive data emerge from studies in which CD4+ cells overexpressing BDNF were injected ICV in 5XFAD mice, resulting in increased neuronal viability and synaptic rescue (Eremenko et al., 2019). Further, BDNF serum levels are increased in preclinical stages of AD (Angelucci et al., 2010; Laske et al., 2011), when compensatory mechanisms are initiated in the attempt to prevent neuronal degeneration (Merlo et al., 2019), and in PBMCs derived from MCI patients, when exposed *ex vivo* to Aβ include CD4+ cells with higher levels of BDNF in comparison to PBMCs derived from AD patients (Baglio et al., 2013).

The crosstalk appears reciprocal since not only astrocytes are capable to modify CD4+ cell functions, but also CD4+ cells modulate astrocytes by reducing their inflammatory response to Aβ, as shown by decreased expression of inflammatory cytokines IL-6 and IL-1β. The role played by astrocytes in the CNS is pleiotropic, as they can support neuronal activity as well as modulate BBB functions in healthy and pathological conditions (Spampinato et al., 2019a). Here, we wondered whether, after their interaction with CD4+ cells, astrocytes may differently affect endothelial properties. This happened to be the case since Aβ failed to induce the reported VEGF up-regulation in astrocytes (Spampinato et al., 2017) in the presence of CD4+ cells. We have previously shown that acting on endothelial cells, astrocytic derived VEGF induces endothelial expression and activity of matrix metalloproteinase (MMP)-9 and subsequent reduction of one of its substrates, the junctional protein claudin-5 (Spampinato et al., 2017). Accordingly, in our study, following exposure of astrocytes to CD4+ cells, we did not detect any change in endothelial claudin-5 expression, thus reinforcing the hypothesis that astrocytic response to Aβ is modified in the presence of CD4+ cells.

Finally, it was established that astrocytes modify the expression of endothelial ICAM-1. In particular, we have previously demonstrated that ICAM-1 is expressed in two different glycoforms, depending on the glycosylation status, and astrocytes in response to Aβ induce the high mannose, low molecular weight, ICAM-1 glycoform (Spampinato et al., 2019b) that is involved in increased migration through the endothelial layer at the BBB (Chacko et al., 2011; Scott et al., 2012). This effect appeared dampened when astrocytes were co-cultured with CD4+ cells, suggesting a series of events by which migrated lymphocytes, after their interaction with astrocytes, can induce changes in BBB function, promoting a negative control mechanism that limits their transmigration through the BBB. Although very speculative, this interpretation let us conclude that initial migration of CD4+ cells through the damaged BBB in AD may trigger, through astrocytes, an auto-limiting outcome that plays as a preventing mechanism to limit further peripheral cell infiltration.

**Figure 5:**
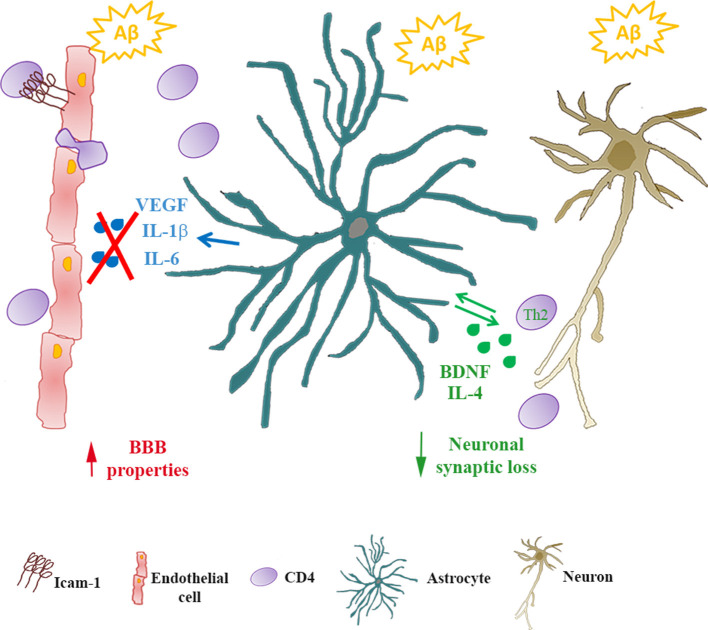
Astrocytes/CD4+ reciprocal crosstalk. In Alzheimer’s disease (AD), infiltrating CD4+ cells cross the BBB reaching the *glia limitans*. Through reciprocal crosstalk, Aβ-exposed astrocytes skew CD4+ towards a Th2 phenotype, inducing the production of IL-4 and BDNF. In the presence of BDNF, Aβ-induced synaptic loss in neurons is prevented. Conversely, in the presence of CD4+ cells, Aβ-exposed astrocytes reduce the release of inflammatory cytokines (IL-6 and IL-1β) and VEGF. As a consequence, endothelial properties at the BBB are preserved.

Data here reported have been observed in *in vitro* models, that only partially represent the complexity of the *in vivo* system. For example, in our experimental setting, we did not take into account the differences between naïve and memory T cells that are known to differ in phenotype, the pattern of migration, responsiveness to antigen and cytokines (Pennock et al., 2013). Although these limitations and the consequent attention that should be used when transferring observations *in vitro* to human pathology, the use of the *in vitro* setting allowed us to analyze the reciprocal crosstalk among peripheral cells, cellular components of the BBB and neurons under conditions of exposure to Aβ. We confirmed that astrocytes, at least in the early phases of AD disease, play a central role in this interaction as they can modify endothelial properties as well as CD4+ cell phenotype and features (Figure 5). As a result, neurons appeared less vulnerable to the effects of Aβ. CD4+ cells, on their side, modified the ability of astrocytes to affect endothelial properties in response to Aβ implying a potential protective effect on the function of the BBB itself.

## Data Availability Statement

All datasets presented in this study are included in the article.

## Author Contributions

All authors gave a significant contribution to the study. SS participated in the study conception and design, carried out the experimental part, and wrote the manuscript. MF and EF participated in selected experimental procedures. YS and TK established and provided the human astrocytic and endothelial cell lines. SM contributed to the interpretation of data and critically revised the manuscript. MS participated in study conception, acquired funding, and reviewed the manuscript.

## Conflict of Interest

The authors declare that the research was conducted in the absence of any commercial or financial relationships that could be construed as a potential conflict of interest.

## Abbreviations

AC: astrocytes
AD: Alzheimer’s disease
Aβ: β-amyloid
BBB: blood-brain barrier
BDNF: brain-derived neurotrophic factor
CD: cluster of differentiation
CNS: central nervous system
ICAM-1: intercellular adhesion molecule 1
IFNγ: interferon γ
IL-4: interleukin 4
MHC: major histocompatibility complex
MW: molecular weight
PBMCs: peripheral blood mononuclear cells
TEM: trans-endothelial migration
Th: T-helper
TJ: tight junctions
TrkB: Tropomyosin receptor kinase B
VCAM-1: vascular cell adhesion molecule 1
VEGF: vascular endothelial growth factor.
